# Skeletal Muscle PGC-1α Is Required for Maintaining an Acute LPS-Induced TNFα Response

**DOI:** 10.1371/journal.pone.0032222

**Published:** 2012-02-27

**Authors:** Jesper Olesen, Signe Larsson, Ninna Iversen, Simi Yousafzai, Ylva Hellsten, Henriette Pilegaard

**Affiliations:** 1 Centre of Inflammation and Metabolism and Copenhagen Muscle Research Centre, Department of Biology, University of Copenhagen, Copenhagen, Denmark; 2 Copenhagen Muscle Research Centre, Department of Sport Sciences, University of Copenhagen, Copenhagen, Denmark; University of Las Palmas de Gran Canaria, Spain

## Abstract

Many lifestyle-related diseases are associated with low-grade inflammation and peroxisome proliferator activated receptor γ coactivator (PGC)-1α has been suggested to be protective against low-grade inflammation. However, whether these anti-inflammatory properties affect acute inflammation is not known. The aim of the present study was therefore to investigate the role of muscle PGC-1α in acute inflammation. Quadriceps muscles were removed from 10-week old whole body PGC-1α knockout (KO), muscle specific PGC-1α KO (MKO) and muscle-specific PGC-1α overexpression mice (TG), 2 hours after an intraperitoneal injection of either 0.8 µg LPS/g body weight or saline. Basal TNFα mRNA content was lower in skeletal muscle of whole body PGC-1α KO mice and in accordance TG mice showed increased TNFα mRNA and protein level relative to WT, indicating a possible PGC-1α mediated regulation of TNFα. Basal p65 phosphorylation was increased in TG mice possibly explaining the elevated TNFα expression in these mice. Systemically, TG mice had reduced basal plasma TNFα levels compared with WT suggesting a protective effect against systemic low-grade inflammation in these animals. While TG mice reached similar TNFα levels as WT and showed more marked induction in plasma TNFα than WT after LPS injection, MKO PGC-1α mice had a reduced plasma TNFα and skeletal muscle TNFα mRNA response to LPS. In conclusion, the present findings suggest that PGC-1α enhances basal TNFα expression in skeletal muscle and indicate that PGC-1α does not exert anti-inflammatory effects during acute inflammation. Lack of skeletal muscle PGC-1α seems however to impair the acute TNFα response, which may reflect a phenotype more susceptible to infections as also observed in type 2 diabetes patients.

## Introduction

The transcriptional coactivator peroxisome proliferator activated receptor γ (PGC)-1α is known to influence many aspects of metabolism and the role of PGC-1α as a master regulator of mitochondrial biogenesis and oxidative metabolism in skeletal muscle (SkM) has been confirmed repeatedly over the last decade [1]–[3]. Recently it has been suggested that PGC-1α also exerts anti-inflammatory effects. Hence overexpressing PGC-1α specifically in SkM reduces an age-associated increase in serum TNFα as well as TNFα mRNA and protein expression in SkM [4]. In accordance TNFα mRNA expression and serum TNFα increases dramatically after a single exercise bout in muscle specific PGC-1α knockout mice, but not in WT [5]. Additionally, a negative correlation has been reported between PGC-1α and TNFα mRNA levels in SkM of type 2 diabetes patients independent of BMI [6]. Taken together, these observations indicate that PGC-1α either directly or through PGC-1α mediated adaptations in SkM provides anti-inflammatory effects that potentially contribute to preventing low-grade inflammation present in many life-style related diseases [7].

Inflammatory mediators have however also been suggested to regulate PGC-1α expression [8]–[10]. Previous findings in human cardiomyocytes indicate that nuclear factor kappa light chain-enhancer of activated B cells (NFκB) binds to and inactivates PGC-1α and that TNFα enhances such NFκB mediated PGC-1α inhibition and the concomitant metabolic effects of PGC-1α [8]. In addition, a recent study shows that lipopolysaccharide (LPS) downregulates PGC-1α expression and reduces fat oxidation in mouse cardiomyocytes and mouse heart in vivo [10]. On the other hand, incubating C2C12 cells with TNFα has been reported to increase PGC-1α dependent transcriptional activity, and LPS treatment in mice has been shown to increase mitochondrial respiration when PGC-1α is elevated in SkM [9]. This suggests that PGC-1α itself is regulated during acute inflammation and these somewhat contradicting observations underline, that the role of PGC-1α in acute inflammation is not fully elucidated. Based on the findings that PGC-1α mediated adaptations in SkM protect against low-grade inflammation, it may be expected that SkM PGC-1α impairs the ability to elicit a robust acute inflammatory response.

An acute inflammatory response is a conserved mechanism highly essential for protection against invading microorganisms in all species. The response involves the induction of both pro- (TNFα, IL-6) and anti-inflammatory (IL-6 and IL-10) cytokines mediated through p38 mitogen-activated protein kinases (p38) as well as NFκB and c-Jun N-terminal kinase (JNK) signaling [11]. LPS treatment is a well-established and frequently used model to induce acute inflammation in humans and rodents [12]–[14]. Although circulating and infiltrating immune cells are seen as the traditional responders during acute inflammation, LPS stimulation has been shown to evoke p38, NFkB and JNK signaling and concomitant production of cytokines like TNFα in many tissues including SkM [9], [14], [15], which likely contributes to systemic levels during acute inflammation. This is supported by the findings that SkM functions as an endocrine organ [16] and that C2C12 cells incubated with LPS have been reported to produce and secrete IL-6 to the medium [15].

The aim of the present study was to investigate whether the level of PGC-1α expression in skeletal muscle affects an acute inflammatory response locally in skeletal muscle and systemically. This was addressed by giving LPS injections to PGC-1α whole body knockout mice (KO), muscle specific PGC-1α knockout mice (MKO) and transgenic mice overexpressing PGC-1α specifically in SkM (TG). In addition, to investigate the potential secretion of TNFα from muscle cells, primary myotubes from mouse skeletal muscle were treated with LPS.

## Materials and Methods

### Ethics Statement

Experiments were approved by the “Animal Experiment Inspectorate” in Denmark (permission number: 2009/561-1607) and complied with the European convention for the protection of vertebrate animals used for experiments and other scientific purposes (Council of Europe, no. 123, Strasbourg, France, 1985).

### Mice

Generation and phenotypes of the whole body PGC-1α KO mice, MKO PGC-1α and TG PGC-1α mice used in the present study have been described elsewhere [1], [17], [18]. Whole body PGC-1α KO mice and littermate WT were obtained by crossbreeding of heterozygous PGC-1α KO parents. MKO and littermate WT were obtained by crossing a +/− Cre, Flox/Flox parent with a Flox/Flox parent, while TG and their littermate WT were obtained by crossbreeding of TG and WT parents. The genotypes of the mice were determined by PCR-based genotyping, as previously described [19]. Mice were kept on a 12∶12 hour light/dark cycle and had access to water and standard rodent chow ad libitum (Altromin no. 1324, Brogården, Lynge, Denmark).

### Experimental protocol

Each group consisted of 10 mice with equal number of male and female mice. Ten weeks old PGC-1α KO, MKO PGC-1α, TG PGC-1α and their respective littermate WT mice were given an intraperitoneal injection of either saline as control or 0.8 µg LPS (Sigma, St Louis, MO, USA) per gram mouse, dissolved in sterile isotonic saline. Based on a preceding pilot study (data not shown) and previous studies [14], [15], [20] showing a pronounced inflammatory plasma and mRNA response 2 hours after LPS treatment, all mice in the present study were euthanized by cervical dislocation 2 hours post injection. Trunk blood was collected after decapitation and quadriceps muscles were quickly removed from all three mouse strains and frozen in liquid nitrogen. In addition visceral adipose tissue and liver were also removed from whole body PGC-1α KO mice. Samples were stored at −80°C until analyzed.

### Primary cell cultures

C57BL/6 mice were euthanized by cervical dislocation and limb skeletal muscles were quickly dissected out and placed in 15 ml ice cold Dulbecco's phosphate buffered saline solution (DPBS; Invitrogen, Carlsbad, CA, USA) containing 1% glucose and 0.5% penicillin/streptomycin (Invitrogen) and placed on ice. Satellite cells were isolated and handled as previously described [21]. One day prior to the experiment, the Fusion Medium (FM) containing DMEM (Invitrogen) with 10% horse serum and 0.1% L-glutamine was exchanged with DMEM without phenol red (Invitrogen) containing 0.5% glucose, 0.05% L-glutamin and 0.1% BSA (DMEM without phenol red). Cells were used for experiments on day 8, where sufficient maturation and differentiation of the myotubes were observed. On the day of the experiment, the cells were washed in DMEM without phenol red. Half of the cell cultures (n = 6, in 3 independent experiments) were treated with 1.0 µg LPS/ml media and the other half was treated with DMEM without phenol red as control. LPS was dissolved in dimethyl sulfoxide (DMSO) and then diluted in DMEM without phenol red. After 2 hours of incubation, the medium was collected and the cells were harvested in Trizol reagent (Invitrogen). The samples were stored at −80°C until analyzed.

### Cell culture media

The TNFα protein content in media from the incubated primary cell cultures was determined by Enzyme-linked immunosorbent assay (ELISA) according to the protocol of the manufacturer (eBioscience, San Diego, CA, USA). The absorption at 450 nm was determined and the TNFα protein content in the medium was calculated based on a standard curve generated from a serial diluted standard.

### Plasma cytokines

Plasma was processed from the blood samples by centrifugation (2600 g for 15 min at 4°C). A MSD multiplex ELISA kit (MesoScaleDiscovery, Gaithersburg, Maryland, USA) pre-coated with antibodies against TNFα, IL-6 and IL-10 was used according to the manufacturer's protocol. MSD plates were measured on a MSD Sector Imager 2400 plate reader. Raw data were measured as electrochemiluminescence signal (light) detected by photodetectors and analyzed using the Discovery Workbench 3.0 software (MSD). A standard curve was generated for each analyte and used to determine the concentration of analytes in each sample.

### RNA isolation and Reverse Transcription

Total RNA was isolated from ∼20–25 mg crushed muscle tissue, liver and adipose tissue with the guanidinium thiocyanate-phenol-chloroform method as previously described [22], [23] and in the cell culture experiment with the Trizol method following the manufacture's guidelines (Invitrogen). The final pellets were re-suspended in DEPC treated H_2_O containing 0.1 mM EDTA. RNA was quantified by measuring the absorbance at 260 nm.

Reverse transcription (RT) was performed using the Superscript II RNase H^−^ system (Invitrogen) as previously described [23] and the cDNA samples were diluted in nuclease-free H_2_O.

### Real-time PCR

Real-time PCR was performed using an ABI 7900 sequence-detection system (Applied Biosystems, Foster City, CA). Primers and TaqMan probes for amplifying gene-specific mRNA fragments were designed using the database from ensemble.org and Primer Express (Applied Biosystems). All TaqMan probes were 5′-FAM and 3′-TAMRA labeled, and primers and Taqman probes were obtained from TAG Copenhagen (Copenhagen, Denmark) except GAPDH (5′-FAM and 3′-TAMRA) and beta-actin (5′-VIC and 3′-nonflourescence), both obtained as a pre-developed assay reagent (Applied Biosystems). The sequences are given in Table 1. Real-time PCR was performed in triplicates in a total reaction volume of 10 µl using Universal Mastermix (Applied Biosystems) except TNFα converting enzyme (TACE), which was amplified using SYBGreen (Applied Biosystems). Cycle threshold (Ct) was converted to a relative amount by use of a standard curve constructed from a serial dilution of a pooled RT sample run together with the samples. For muscle samples, target gene mRNA content was for each sample normalized to single-stranded cDNA content determined by OliGreen reagent (Molecular Probes, Leiden, The Netherlands) as previously described [24]. For liver and cell culture samples, target gene mRNA content was normalized to beta-actin mRNA, which was unaffected by genotype and treatment in the respective samples and for adipose tissue samples, target gene mRNA content was normalized to GAPDH mRNA, which was unaffected by genotype and treatment in these samples.

**Table 1:** Primer and Taqman probe sequences.

	Forward primers	Reverse primers	Taqman Probes
**TNFα**	5′ ATGGCCCAGACCCTCACA 3′	5′ TTGCTACGACGTGGGCTACA 3′	5′ TCAGATCATCTTCTCAAAATTCGAGTGACAAGC 3′
**IL-6**	5′ GCTTAATTACACATGTTCTCTGGGAAA 3′	5′ CAAGTGCATCATCGTTGTTCATAC 3′	5′ ATCAGAATTGCCATTGCACAACTCTTTTCTCAT 3′
**IL-10**	5′ AGAGAAGCATGGCCCAGAAAT 3′	5′ CAGGGGAGAAATCGATGACA 3′	5′ CAGGGGAGAAATCGATGACA 3′
**TACE**	5′ TGCAAGGCTGGGAAATGC 3′	5′ TTG CACGAGTTGTCAGTGTCAA 3′	
**TLR4**	5′ TCTGATCATGGCACTGTTCTTCTC 3′	5′ CTGATCCATGCATTGGTAGGTAATATTA 3′	5′ CAGGAAGCTTGAATCCCTGCATAGAGGTAGTTC 3′
**F4/80**	5′ GGCTGCCTCCCTGACTTTC 3′	5′ TGCACTGCTTGGCATTGC 3′	5′ TCCTTTTGCAGTTGAAGTTTCCATATCCTTGG 3′

Sequences for forward and reverse primers as well as Taqman probes used. Tumor necrosis factor (TNF)α, interleukin (IL)-6, IL-10, TNFα converting enzyme (TACE), toll-like receptor 4 (TLR4), macrophage specific marker (F4/80).

### Muscle lysate

Crushed ∼20–25 mg quadriceps muscles were homogenized in an ice-cold buffer (10% Glycerol, 20 mM Na-pyrophosphate, 150 mM NaCl, 50 mM Hepes, 1% NP-40, 20 mM β-glycerophosphate, 10 mM NaF, 1 mM EDTA, 1 mM EGTA, 2 mM PMSF, 10 µg/ml Aprotinin, 10 µg/ml Leupeptin, 2 mM Na_3_VO_4_, 3 mM Benzamidine, pH 7.5) for 1 min using a tissuelyser (TissueLyser II; QIAGEN, Germany) with 30 oscillations per second. Homogenates were rotated end over end for 30 min at 4°C. The procedure with the tissuelyser and the end over end rotation was repeated. Lysates were generated by centrifugation at 16,000 g for 20 min at 4°C and collection of the protein supernatant (lysate). Protein content in lysates was measured by the bicinchoninic acid method (Thermo Scientific, Rockford, IL, USA).

### SDS-PAGE and western blotting

TNFα protein as well as phosphorylation of p38^Thr180, Tyr182^ and p65^Ser536^, the active subunit of NFκB, was measured in muscle lysates by SDS-PAGE (10% or 15% Tris-HCl gel, BioRad, Denmark) and western blotting using PVDF membrane and semi-dry transfer. Twentyfive µg protein lysate was loaded for TNFα and 15 µg protein lysate for p65 and p38 phosphorylation. After the transfer, the PVDF membrane was blocked for 1 h at room temperature (TBST+5% bovine serum albumin (BSA)) and then incubated with primary antibody (1∶1000 in TBST+5% BSA) over night. Commercially available antibodies were used to detect TNFα (Cell Signaling Technology #3707), p38^Thr180, Tyr182^ (Cell Signaling Technology #4511) and p65^Ser536^ (Cell Signaling Technology #3033). The following day, the membrane was incubated with horseradish peroxidase-conjugated secondary antibody (Dako, Denmark) for 1 h at room temperature (1∶5000 in TBST+5% BSA). Immobilon Western (Millipore Corporation, MA) was used as detection system and bands were visualized using an Eastman Kodak Co. Image Station 2000 MM. Band intensity was quantified using Kodak Molecular Imaging Software v. 4.0.3, and protein content or phosphorylation was expressed as arbitrary units relative to control samples loaded on each site of each gel.

### Statistics

Two-way ANOVA was used to test the effect of LPS and genotype within each mouse strain. Student Newman Keuls *post hoc* test was used to locate differences. A T-test was used to test if there was any difference in the basal levels between genotypes within each mouse strain. All values are presented as means ± S.E. A P<0.05 was considered significant.

## Results

### LPS-induced TNFα responses in primary myotubes

To verify that skeletal muscle produces and secretes TNFα in response to LPS, primary myotubes isolated from C57BL/6 mice were incubated with LPS. LPS treatment increased (p<0.05) TNFα mRNA ∼30 fold and TNFα protein in the media increased (p<0.05) markedly from non-detectable levels in control cells to ∼400 pg/ml (data not shown).

### Plasma cytokines

#### Plasma TNFα

**Figure 1:**
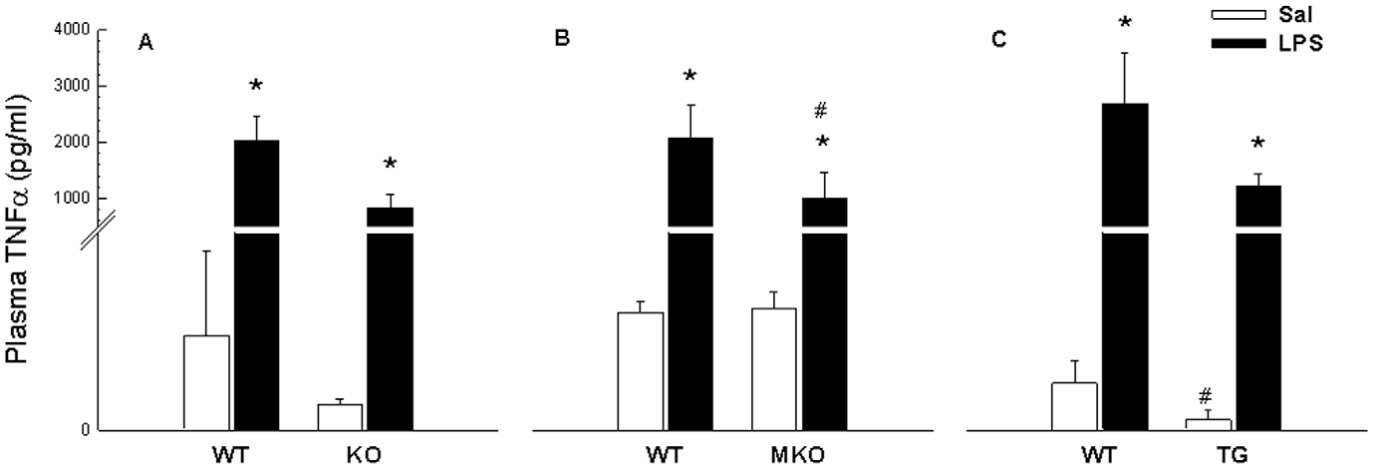
Plasma TNFα. Plasma tumor necrosis factor (TNF)α from whole body PGC-1α knockout (KO) (A), muscle specific PGC-1α KO (MKO) (B) and muscle specific PGC-1α overexpression (TG) mice (C) and their respective littermate wild type (WT) mice, 2 hours after injection with either saline (Sal) as control or lipopolysaccharide (LPS). Values are presented as means ± S.E. with n = 6–10 in each group, except WT of whole body KO strain injected with saline where n = 3, due to lack of blood. *: Significantly different from Sal within given genotype, p<0.05. #: Significantly different from WT within given treatment, p<0.05. Notice the bi-sected y-axis.

TG mice overexpressing PGC-1α specifically in skeletal muscle had reduced basal plasma TNFα level (0.6±0.5 pg/ml) compared with WT (2.6±1.2 pg/ml), whereas there was no difference either in whole body PGC-1α KO or in MKO PGC-1α mice compared with WT (fig. 1). In all groups, LPS increased (p<0.05) the plasma TNFα level. Whereas there was no genotype difference in the LPS-induced plasma TNFα response between whole body PGC-1α KO mice and WT, MKO PGC-1α mice had a reduced (p<0.05) response to LPS compared with WT. Furthermore, as a consequence of the lowered plasma TNFα level in TG PGC-1α mice in the basal state, a statistical interaction was observed between TG PGC-1α mice and WT. Thus the LPS-induced plasma TNFα response was higher (∼2000 fold) in TG PGC-1α mice than in WT mice (∼1000 fold).

#### Plasma IL-6

Basal plasma IL-6 levels were unaffected by genotype. LPS induced a ∼100–400 fold increase (p<0.05) in plasma IL-6 in all groups with no difference between genotypes (table 2).

**Table 2:** Plasma IL-6 and IL-10.

	Whole body PGC-1α KO strain				PGC-1α MKO strain				TG PGC-1α strain			
	WT	WT	KO	KO	WT	WT	MKO	MKO	WT	WT	TG	TG
	Sal	LPS	Sal	LPS	Sal	LPS	Sal	LPS	Sal	LPS	Sal	LPS
IL-6 (pg/ml)	530±404	51053±7556*	213±42	37245±10319*	437±45	133278±25529*	490±55	132587±35835*	485±142	63907±801*	265±48	63253±2346*
IL-10 (pg/ml)	147±30	4496±412*	152±43	3186±672*	177±10	4744±762*	203±26	4057±1208*	201±37	5670±527*	142±24	3377±519*

Plasma interleukin (IL)-6 and IL-10 from whole body PGC-1α knockout (KO), muscle specific PGC-1α KO (MKO) and muscle specific PGC-1α overexpression (TG) mice and their respective littermate wild type (WT) mice, 2 hours after injection with either saline (Sal) as control or lipopolysaccharide (LPS). Values are presented as means ± S.E. with n = 6–10 in each group, except WT of whole body KO strain injected with saline where n = 3, due to lack of blood.

*: Significantly different from Sal within given genotype, p<0.05.

#### Plasma IL-10

Basal plasma IL-10 levels were unaffected by genotype. LPS induced a ∼20–30 fold increase (p<0.05) in plasma IL-10 in all groups with no difference between genotypes (table 2).

### Basal and LPS-induced mRNA levels in skeletal muscle

#### TNFα mRNA

**Figure 2:**
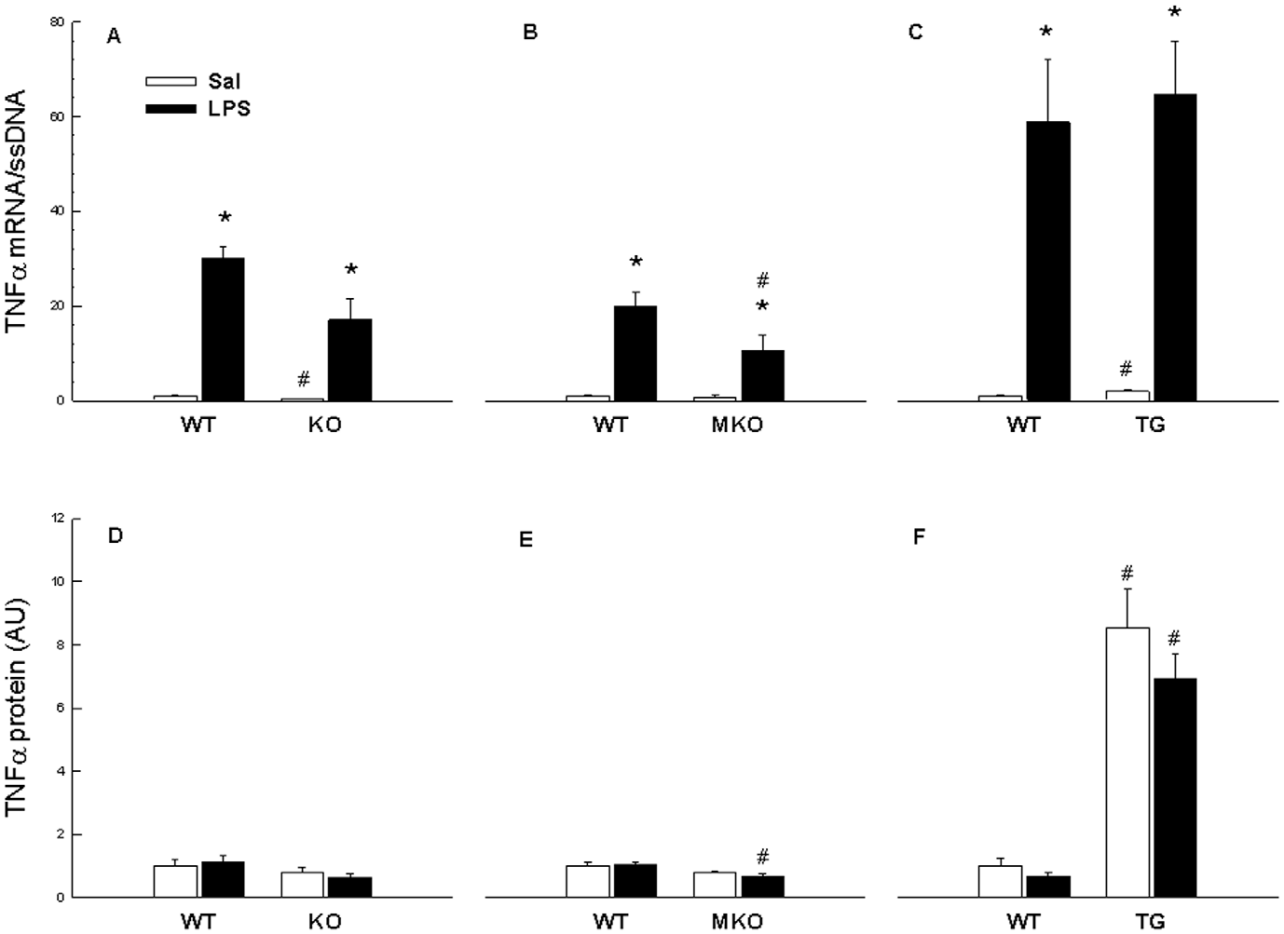
TNFα mRNA and protein content in quadriceps. Tumor necrosis factor (TNF)α mRNA and protein in quadriceps muscle from whole body PGC-1α knockout (KO) (A,D), muscle specific PGC-1α KO (MKO) (B,E) and muscle specific PGC-1α overexpression (TG) mice (C,F) and their respective littermate wild type (WT) mice, 2 hours after injection with either saline (Sal) as control or lipopolysaccharide (LPS). Values are presented as means ± S.E. with n = 10 in each group. *: Significantly different from Sal within given genotype, p<0.05. #: Significantly different from WT within given treatment, p<0.05.

In the basal state, whole body PGC-1α KO mice had ∼60% lower (p<0.05) TNFα mRNA content in skeletal muscle than WT and in line with this, TG mice had ∼2 fold higher (p<0.05) TNFα mRNA content than WT, whereas no significant difference was detected between MKO and WT despite a visual ∼20% reduction in MKO. LPS injections increased (p<0.05) the TNFα mRNA level ∼13–60 fold in all groups. While no genotype differences were observed in the LPS response in the whole body KO and TG strains, the TNFα mRNA level after LPS treatment was in MKO mice ∼50% lower (p<0.05) than in WT (fig. 2).

#### TACE mRNA

To further examine the regulation of TNFα, a metalloproteinase, TACE, which cleaves the membrane-associated precursor of TNFα to the soluble and biological active form of TNFα, was analyzed. In general, MKO mice had 30–40% lower (p<0.05) TACE mRNA levels in SkM than WT mice, both at basal conditions and after LPS injections. No difference was observed in whole body PGC-1α KO or TG mice relative to WT. LPS had no effect on TACE mRNA levels in any of the strains (table 3).

**Table 3:** Skeletal muscle mRNA and phosphorylation levels.

	Whole body PGC-1α KO strain				PGC-1α MKO strain				TG PGC-1α strain			
	WT	WT	KO	KO	WT	WT	MKO	MKO	WT	WT	TG	TG
	Sal	LPS	Sal	LPS	Sal	LPS	Sal	LPS	Sal	LPS	Sal	LPS
TACE mRNA	1±0.1	1.1±0.1	0.9±0.1	1.1±0.2	1±0.2	1±0.2	0.6±0.2#	0.7±0.1#	1±0.04	1.1±0.1	1.1±0.1	1.2±0.1
TLR4 mRNA	1±0.1	1±0.1	1±0.1	1.2±0.1	1±0.2	1±0.2	0.6±0.2	0.9±0.2	1±0.1	0.9±0.1	0.9±0.1	0.8±0.1
IL-6 mRNA	1±0.3	213±45*	1.7±0.5	231±47*	1±0.2	230±53*	0.5±0.1#	183±70*	1±0.2	130±25*	0.6±0.2	129±40*
IL-10 mRNA	1±0.1	4.8±0.4*	0.4±0.1#	2.5±0.4*	1±0.3	2.2±0.5*	0.4±0.1	2.0±0.4*	1±0.1	2.9±0.3*	1±0.2	2.5±0.5*
F4/80 mRNA	1±0.1	1.0±0.1	1.1±0.2	0.9±0.2	1±0.3	0.9±0.2	0.4±0.2	0.8±0.2	1±0.1	1.01±0.1	1.1±0.1	0.7±0.1
p38-p (AU)	1±0.2	1.3±0.2	1.2±0.2	1.6±0.1	1±0.3	1±0.2	0.4±0.04	1.0±0.1	1±0.1	1.5±0.1*	0.7±0.1	1.6±0.2*

TNFα converting enzyme (TACE), toll-like receptor 4 (TLR4), interleukin (IL)-6, IL-10, macrophage specific marker (F4/80) mRNA as well as p38^Thr180, Tyr182^ phosphorylation (p38-p) in quadriceps muscle from whole body PGC-1α knockout (KO), muscle specific PGC-1α KO (MKO) and muscle specific PGC-1α overexpression (TG) mice and their respective littermate wild type (WT) mice, 2 hours after injection with either saline (Sal) as control or lipopolysaccharide (LPS). Values are presented as means ± S.E. with n = 10 in each group.

*: Significantly different from Sal within given genotype, p<0.05.

# : Significantly different from WT within given treatment, p<0.05.

#### TLR4 mRNA

There were no differences in either basal TLR4 mRNA expression or after LPS injections in any of the strains (table 3).

#### IL-6 mRNA

MKO mice had ∼50% lower (p<0.05) basal SkM IL-6 mRNA level than WT, whereas no genotype differences were observed in the basal IL-6 mRNA level in the whole body KO or TG mouse strain. In all groups, LPS induced a 130–400 fold increase (p<0.05) in IL-6 mRNA with no genotype differences (table 3).

#### IL-10 mRNA

In the basal state, whole body PGC-1α KO mice had ∼55% lower (p<0.05) SkM IL-10 mRNA level than WT, whereas there were no significant genotype differences in the MKO (despite a visual 57% reduction) or TG strains. LPS increased (p<0.05) the IL-10 mRNA content ∼2–6 fold in all groups with no significant difference between genotypes (table 3).

#### F4/80 mNRA

There were no genotype differences in the mRNA level of the macrophage specific marker, F4/80, in SkM of any of the strains and LPS had no effect on the F4/80 mRNA level, indicating no LPS-induced recruitment of macrophages to skeletal muscle 2 hours after LPS treatment (table 3).

### TNFa protein in skeletal muscle

#### TNFα protein content

TG mice had ∼9 fold higher (p<0.05) basal SkM TNFα protein level relative to WT, while there were no genotype differences in whole body KO and MKO PGC-1α mouse strains at basal conditions. LPS did not alter SkM TNFα protein content in any mouse strain, but MKO mice treated with LPS had ∼30% lower (p<0.05) TNFα protein than WT treated with LPS (fig. 2).

### Basal and LPS-induced intracellular signaling in skeletal muscle

#### Phosphorylation of p65^ser536^

**Figure 3:**
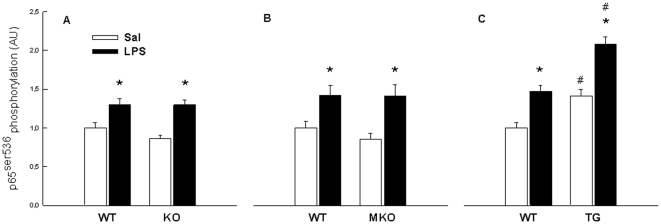
Phosphorylation of p65. Phosphorylation of p65^ser536^ in quadriceps muscle from whole body PGC-1α knockout (KO) (A), muscle specific PGC-1α KO (MKO) (B) and muscle specific PGC-1α overexpression (TG) mice (C) and their respective littermate wild type (WT) mice, 2 hours after injection with either saline (Sal) as control or lipopolysaccharide (LPS). Values are presented as mean s± S.E. with n = 10 within each group. *: Significantly different from Sal within given genotype, p<0.05. #: Significantly different from WT within given treatment, p<0.05.

**Figure 4:**
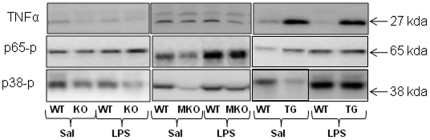
Representative blots. Representative blot of tumor necrosis factor (TNF)α protein, phosphorylation of p65 protein (p65-p) and phosphorylation of p38 (p38-p) in quadriceps muscle from whole body PGC-1α knockout (KO), muscle specific PGC-1α KO (MKO) and muscle specific PGC-1α overexpression (TG) mice and their respective littermate wild type (WT) mice, 2 hours after injection with either saline (Sal) as control or lipopolysaccharide (LPS).

Basal p65 phosphorylation was not affected by genotype in the whole body PGC-1α KO and MKO strains, but notably TG mice overexpressing PGC-1α in skeletal muscle had ∼40% higher (p<0.05) basal SkM p65 phosphorylation than WT mice. This may explain the elevated resting TNFα mRNA and protein level in these mice. LPS increased (p<0.05) p65 phosphorylation ∼30–70% in all three mouse strains. The TG mice reached ∼40% higher (p<0.05) p65 phosphorylation level after LPS treatment than WT, whereas there were no differences between whole body PGC-1α KO or MKO mice and their corresponding WT (fig. 3 and 4).

#### Phosphorylation of p38^Thr180, Tyr182^

LPS induced a similar ∼2 fold increase (p<0.05) in p38 phosphorylation in TG and WT mice, whereas no significant LPS-induced p38 phosphorylation was observed in the whole body PGC-1α KO or the MKO PGC-1α strain. No genotype differences were observed in the LPS-induced p38 phosphorylation in SkM in any of the strains (table 3).

### Basal and LPS-induced mRNA levels in liver and adipose tissue from whole body PGC-1α KO mice

To examine whether PGC-1α regulates the response to acute LPS-induced inflammation in other inflammatory tissues, liver and adipose tissue mRNA was analyzed from whole body PGC-1α KO mice and WT.

#### TNFα and IL-6 mRNA content

No genotype differences were observed in TNFα and IL-6 mRNA in liver or adipose tissue in the basal state. LPS induced a 6–89 fold increase (p<0.05) in TNFα and IL-6 mRNA in liver and adipose tissue with no difference between WT and KO mice (table 4).

**Table 4:** mRNA expression in adipose tissue and liver from whole body PGC-1α KO mice.

	Adipose tissue				Liver			
	WT	WT	KO	KO	WT	WT	KO	KO
	Sal	LPS	Sal	LPS	Sal	LPS	Sal	LPS
TNFα mRNA	1±0.4	12.2±2.1*	1.3±0.4	7.6±1.0*	1±0.2	36.2±3.5*	1.2±0.2	29.3±2.9*
TACE mRNA	1±0.2	2.2±0.6	1.3±0.2	1.0±0.2	1±0.2	1.2±0.1	0.9±0.1	1.0±0.1
IL-6 mRNA	1±0.3	38.8±7.2*	1.7±1.2	22.6±7.1*	1±0.3	88.8±9.7*	1.6±0.5	63.4±10.5*
IL-10 mRNA	1±0.2	4.0±0.9*	1.2±0.2	1.8±0.3	1±0.4	6.7±1.3*	1.0±0.2	5.0±0.6*
F4/80 mRNA	1±0.2	1.5±0.4	1.1±0.2	0.4±0.1	1±0.1	0.9±0.1	1.0±0.2	0.8±0.1

Tumor necrosis factor (TNF)α, TNFα converting enzyme (TACE), interleukin (IL)-6, IL-10, macrophage specific marker (F4/80) mRNA in adipose tissue and liver from whole body PGC-1α knockout (KO) and WT mice, 2 hours after injection with either saline (Sal) as control or lipopolysaccharide (LPS). Values are presented as means ± S.E. with n = 10 in each group.

*: Significantly different from Sal within given genotype, p<0.05.

#### TACE mRNA content

There was no genotype difference in TACE mRNA level in the liver or the adipose tissueat basal conditions and no changes with LPS were observed (table 4).

#### IL-10 mRNA content

In the basal state, no genotype differences were observed in IL-10 mRNA in either the liver or adipose tissue. LPS increased (p<0.05) the IL-10 mRNA level in the liver similarly in KO and WT mice. In the adipose tissue, IL-10 mRNA only increased significantly (p<0.05) in WT, whereas only visual increase (p = 0.094) were observed in PGC-1α KO mice in response to LPS treatment (table 4).

#### F4/80 mRNA content

No genotype differences were observed in F4/80 mRNA content in either the liver or adipose tissue in the basal state. F4/80 mRNA was unaffected by LPS treatment in both liver and adipose tissue from WT and KO mice (table 4).

## Discussion

The main findings of the present study are that whole body PGC-1α KO mice had a reduced basal TNFα mRNA level and that mice overexpressing PGC-1α in skeletal muscle increased the basal TNFα mRNA and protein content in skeletal muscle, suggesting a PGC-1α mediated regulation of TNFα expression in skeletal muscle. However, basal plasma TNFα was reduced in mice overexpressing PGC-1α suggesting diminished secretion of TNFα and indicating that high TNFα levels in SkM do not lead to systemic low-grade inflammation. In addition, while high PGC-1α levels in skeletal muscle elicited a more marked LPS-induced plasma TNFα response, muscle specific knockout of PGC-1α resulted in a lower LPS-induced plasma TNFα and TNFα mRNA response, potentially reflecting a phenotype more susceptible to infections.

By use of three different PGC-1α mice models, the present study provides new insight to the role of SkM PGC-1α in basal and acute inflammation. The present findings that LPS treatment resulted in similar TNFα, IL-6 and IL-10 levels in skeletal muscle and plasma in TG and WT mice provide evidence that high levels of PGC-1α in SkM do not alter the level of inflammatory markers during acute inflammation. Of notice is however, that although the TG PGC-1α mice had a lower basal plasma TNFα level, LPS elicited a more marked fold increase in these mice. In accordance, the reduced LPS-induced TNFα response in MKO PGC-1α mice both systemically and in skeletal muscle indicates that lack of PGC-1α or PGC-1α mediated metabolic adaptations impair the ability to respond to acute inflammation. Together this suggests that high SkM PGC-1α does not exert anti-inflammatory effects during acute inflammation and SkM PGC-1α is required for a normal acute inflammatory response. Interestingly the observed responses in PGC-1α MKO and to some extent whole body PGC-1α KO mice, resemble the previously reported attenuations in LPS-induced plasma TNFα response in type 2 diabetes patients [25]. This may suggest that low levels of PGC-1α in skeletal muscle, as observed in physically inactive subjects [26] and in type 2 diabetes patients [27], impair the inflammatory response to invading pathogens.

TLR4 is the main receptor mediating LPS-induced responses in SkM. The observed similar levels of TLR4 mRNA independent of genotype and treatment indicates, that the observed lower plasma TNFα and SkM TNFα mRNA in MKO PGC-1α mice than WT in the present study are not due to a difference at the receptor level. The observed LPS-induced increase in phosphorylation of the active subunit of NFκB, p65, and to some extent p38 signaling supports that both NFκB and p38 contributed to the inflammatory response after LPS treatment in the present study. However, lack of genotype differences in the LPS-induced changes in signaling indicates that neither NFκB (p65 phosphorylation) nor p38 signaling can explain the attenuated responses in plasma TNFα and TNFα mRNA in SkM observed in MKO PGC-1α mice. Differences in NFκB or p38 signaling, earlier than 2 hour after LPS injections, of course can not be ruled out. The more marked fold increase in plasma TNFα in TG mice upon LPS treatment may on the other hand be linked to the higher p65 phosphorylation level in TG than WT. However, the unaffected TNFα protein content in TG mice in response to LPS is as such in accordance with such a mechanism as more secretion could keep the increased level constant. But the similar TNFα mRNA response in TG and WT does not support that a difference in NFκB signaling alone is responsible for the more marked plasma TNFα response in TG mice. As the LPS-induced SkM IL-6 and IL-10 mRNA and plasma levels are independent of genotype, the regulatory mediators and signaling factors involved in eliciting the observed TNFα genotype difference in MKO PGC-1α mice in response to LPS injections appear to be TNFα specific. Interestingly, the finding that MKO PGC-1α mice, both in the basal state and in response to LPS, had reduced SkM mRNA expression of the TNFα regulatory enzyme TACE [28], [29] indicates that these mice may have a decreased ability to cleave TNFα into its biological active form and thereby a reduced secretion of TNFα protein from SkM relative to WT. Such a mechanism may at least to some extent contribute to the observed impaired LPS-induced plasma TNFα response when PGC-1α is lacking in SkM.

The findings that whole body PGC-1α KO mice had lowered basal TNFα mRNA level and that TG mice had elevated basal TNFα mRNA and protein level in skeletal muscle compared with WT are in contrast to previous observations in 22 months old TG PGC-1α mice [4] and young MKO PGC-1α mice [6]. The elevated TNFα levels in skeletal muscle of TG mice in the present study may be explained by the observed higher level of basal p65 phosphorylation in skeletal muscle of TG mice. NFκB is activated by several different stress associated stimuli such as LPS and TNFα [30], but is also known to be a redox-sensitive transcription factor. An increased basal p65 activity in TG mice could possibly be due to an imbalance in ROS production and antioxidant defense, which could lead to an altered redox state in the mitochondria of these mice. However, based on enhanced superoxide dismutase 2 protein content in skeletal muscle of TG PGC-1α mice in a previous [4] and the present study (data not shown) as well as reduced protein carbonylation and oxidized nucleic acids in old TG PGC-1α mice compared with age-matched controls [4], there are no indications of elevated ROS in SkM muscle of TG PGC-1α overexpression mice. Thus, increased mitochondrial ROS levels do not seem to be the explanation for the elevated basal p65 phosphorylation and the mechanism responsible for the enhanced p65 phosphorylation in SkM of TG mice remains to be determined.

The finding that TG PGC-1α mice had elevated SkM TNFα mRNA and protein and reduced plasma TNFα relative to WT in the basal state, while there were no genotype differences in SkM and plasma for IL-6 and IL-10, again indicates a TNFα specific regulation. As TACE is required for the conversion of the TNFα precursor to circulating TNFα, a specific regulation of TACE could potentially have contributed to the observed PGC-1α dependent difference. However, the observed similar TACE mRNA levels in SkM of WT and TG mice indicate that the amount of TACE is not responsible for the observed differences between TNFα expression in SkM and systemic levels in TG mice in the basal state, although the present study cannot elucidate whether the activity of TACE is altered in these mice. Another possible explanation for the discrepancy could be that the contribution from skeletal muscle plays a minor role regarding systemic plasma TNFα levels, because TNFα released from skeletal muscle is diluted in the circulation. Cytokine production is usually believed to originate from traditional immune cells like macrophages, lymphocytes and monocytes in the blood stream and infiltrated in various tissues. Adipose tissue and the liver are also both known to respond to acute inflammation, which is also supported by the present increases in TNFα, IL-6 and IL-10 mRNA in liver and adipose tissue in response to LPS. But as skeletal muscle is the largest organ of the body and previously has been shown to produce cytokines [14], [31], [32], it may be speculated that skeletal muscle plays a significant overall role during acute inflammation. The present observation that primary myotubes incubated with LPS produced and secreted TNFα supports that skeletal muscle may be an important endocrine tissue during acute inflammation and suggests that skeletal muscle could be classified as an immunological tissue. In addition, the findings that the mRNA level of the macrophage specific marker F4/80 was unchanged with LPS treatment, suggest that the inflammatory response does not originate from newly recruited macrophages but rather resident macrophages or myofibers as suggested from the cell culture results. The magnitude by which SkM contributes to the circulating cytokine level after LPS treatment is difficult to assess and additional studies are needed to elucidate this aspect.

The present finding that overexpression of PGC-1α in skeletal muscle decreased the systemic plasma TNFα level supports a previous study showing that 22 months old TG PGC-1α mice were protected against an age-associated increase in serum TNFα [4]. Because elevated systemic TNFα has been associated with obesity [33] and type 2 diabetes [34] and TNFα is known to induce insulin resistance in vitro [35] and in vivo [36], the lowered plasma TNFα in TG mice would therefore be expected to have beneficial effects. Such a potential ability of high SkM PGC-1α to protect against systemic low-grade inflammation and at the same time posesses the ability to elicit a robust acute inflammatory response underlines that different molecular mechanism mediate these processes. Still, it may be noted, that no previous studies have reported improvements in insulin sensitivity in young TG PGC-1α mice and in fact when fed a high-fat diet for 3 weeks, TG PGC-1α mice become insulin resistant compared with WT controls [37]. In addition, the basal plasma TNFα level in the present study was relatively low (<3 pg/ml) in both TG and WT mice and therefore, the observed genotype difference in plasma TNFα may not necessarily result in metabolic differences at this age.

Finally, to elucidate the potential role of PGC-1α in the acute inflammatory response in other tissues than skeletal muscle, the liver and adipose tissue of whole body PGC-1α KO mice were examined. The observed similar LPS-induced TNFα, IL-6 and IL-10 mRNA responses in liver and adipose tissue from whole body PGC-1α KO mice and WT mice does not indicate that PGC-1α is necessary for a full inflammatory response in these tissues.

In conclusion, PGC-1α seems to be important for basal TNFα expression in skeletal muscle, potentially via effects on p65 phosphorylation. While mice overexpressing PGC-1α in SkM showed more marked plasma TNFα induction and reaching similar TNFα levels after LPS injection as WT, lack of PGC-1α and/or lack of PGC-1α mediated metabolic regulation in skeletal muscle impair a LPS-induced TNFα response both systemically and in skeletal muscle. This suggests that low levels of SkM PGC-1α could lead to an inflexible phenotype with impaired ability to cope with infections. Whether such PGC-1α associated effects are related to direct effects of PGC-1α or are secondary effects of PGC-1α mediated metabolic regulation remains to be determined.
